# The protective role of resilience and social support against burnout during the COVID-19 pandemic

**DOI:** 10.3389/fpubh.2024.1374484

**Published:** 2024-04-30

**Authors:** Shazana Shahwan, Eng Hong Tay, Saleha Shafie, Yoke Boon Tan, Savita Gunasekaran, Rachel Hsiao Shen Tan, Pratika Satghare, Yunjue Zhang, Peizhi Wang, Sing Chik Tan, Mythily Subramaniam

**Affiliations:** Research Division, Institute of Mental Health, Singapore, Singapore

**Keywords:** resilience, social support, burnout, pandemic, stress

## Abstract

**Background:**

The COVID-19 pandemic brought on a range of stressors in homes and workplaces. With no sign of ending after one year, burnout was a concern. Resilience has been known to shield against the effects of stress. While often thought of as an individual trait, previous studies have shown social support to improve resilience. The study aimed to examine the extent of burnout in the Singapore population and whether social support and resilience cushioned the impact of COVID-19 related stressors a year into the pandemic.

**Methods:**

Participants were 858 Singapore residents who participated in a larger study between October 2021 and September 2022. The Copenhagen Burnout Inventory provided Work-and Personal-related burnout scores. Multivariable linear regression was used to identify demographic variables associated with burnout. Path analysis revealed the associations between COVID-19 stressors, social support, resilience and burnout.

**Results:**

22 and 19% of the sample reported work and personal burnout respectively, with younger adults being more burnt out than older adults. Higher stress was associated with higher burnout and higher social support was associated with lower burnout. Path analysis revealed that the relationship between social support and burnout was partially accounted for by increased resilience.

**Conclusion:**

Managing altered work arrangements, career expectations, and increased responsibilities at home may contribute to greater levels of burnout in the younger adults. Increased employer support and targeted interventions could mitigate the impact of these stressors. The study also highlighted the importance of maintaining social connections even while physically distancing.

## Introduction

The COVID-19 outbreak was declared a Public Health Emergency of International Concern (PHEIC) on 30 January 2020 by the World Health Organisation, retaining its status for three years till the declaration was lifted on 5 May 2023. In the initial weeks after COVID-19 was first detected in countries around the globe, citizens were engulfed in panic and trepidation as news of spikes in the number of new cases, hospitalisation rates and death tolls flooded the media. Hospitals were overburdened and medical resources ran low. In order to mitigate transmission of the virus and prevent medical systems from reaching breaking point, stringent physical distancing policies such as school and workplace closures, travel restrictions, quarantine, gathering size limits and at the forefront of these, lockdown was imposed in many countries.

These containment measures would have severe economic, health and social impacts. Governments were faced with difficult trade-offs among these factors (1). As regulations were adjusted with the evolving situation, civilians and institutions altered their routines and operational procedures to comply with them. In addition to the need to acclimatise to the new regulations and the dynamics it brought, individuals battled with the constant negative presence of COVID-19 on media, prolonged social deprivation and other stressors that had no foreseeable end in sight, taxing them physically and mentally, causing burnout over time (2, 3).

The concept of burnout was defined by Maslach and Jackson as a psychological syndrome characterised by emotional exhaustion, feelings of cynicism or depersonalisation and reduced personal accomplishment (4). Majority of the research that examined pandemic burnout focused on frontline healthcare workers who faced the harshest impact of this crisis attributed to long working hours without adequate rest, torment from having to make life-and-death decisions hastily, and pain of losing their patients and colleagues (5). Where burnout was originally conceptualised in the workplace context, it has expanded to other chronically stressful situations (6).

Within homes, burnout was recognised to be heightened among young parents who were struggling with full-time parenting and home-schooling while simultaneously working from home (7). Those who were not parents also reported struggles with work–family conflict, in the form of increased interruptions and distractions due to the presence of family and additional household responsibilities. Adding to that burden, there were expectations—whether real, perceived, or self-imposed—regarding one’s availability to respond quickly during remote working as individuals were assumed to be confined to the home. Combined with the impression that saved commuting time could be used to extend work hours, workers found themselves under increased pressure (8).

High levels of burnout have been shown to be significantly associated with depression, anxiety, and insomnia (9). It has also been associated with weakened immune functioning (10). While some individuals suffer the impact of the pandemic more acutely, others appear to cope better. According to the Transactional Model of Stress, individuals respond to the same stressor differently due to individual characteristics and contextual factors (11). Resilience is an individual response to adverse situations that has been found to mitigate the detrimental effects of stress on burnout (12). Resilience refers to the “ability to bounce back quickly” from highly distressing events (2). Smith et al. proposed that resilience involves confronting (rather than avoiding or denying) the stressful event, orienting oneself towards a future positive outcome of the event and actively engaging in efforts to cope with it (13). As an example, a study among emergency response workers during the second COVID-19 wave in Italy showed that problem-focused coping, being able to get past negative feelings and a strong sense of purpose appeared to offer workers protection against burnout (14).

A contextual factor that has been found to buffer the negative effects of stress from adverse life events on mental and physical health is social support (15). Nitsche et al. reported that greater social connectedness during the lockdown period was associated with lower levels of perceived stress as well as general and COVID-19 specific worries (16). According to the stress-buffering hypothesis, the more social support or resources a person has or perceives to have available, the more likely the individual is to feel in control of the stressful situation. Perceived social support has been theorised to prevent a situation from being appraised as highly stressful whereas received support has been theorised to cushion the impacts of stress by assisting with coping (17). Several studies have in turn shown that social support can be a key mechanism in bolstering resilience (18, 19). Conversely, individuals who lacked social interaction were hypervigilant to threats and had higher negative appraisal of threats resulting in increased overall stress (17).

Singapore is a small metropolitan city-state measuring 728.6 km^2^ with a population of 5.64 million (20). It is regarded as one of the medical hubs in the Asia Pacific region (21) and billed itself as an efficient business city with one of the busiest shipping ports in the world (22). While Singapore’s healthcare system remained resilient during the pandemic and its nationwide vaccination campaign was a success with 80% of the population being fully vaccinated by the end of August 2021, a survey across six Asian countries stated that it was the only country to report burnout as the leading factor affecting mental health of residents during the pandemic (3). This contrasted with Japan, South Korea, and Hong Kong whose residents were most affected by public measures to keep the pandemic under control such as mask-wearing and travel restrictions, while Malaysia and Indonesia were most affected by financial burden due to income loss (3). The uniqueness of this finding to Singapore could be a reflection of the competitive and efficiency-driven culture which was embodied even amidst the pandemic.

The aims of this study were to identify the extent of burnout in the population, sociodemographic correlates of the population that were most affected by burnout and understand the relationships between COVID-19 related stress, burnout, resilience, and social support. Based on Lazarus and Folkman’s Transactional Model of Stress and earlier studies that demonstrated the associations between stress, burnout and resilience (23–25), we hypothesized that (i) greater perceived COVID-19 stressors predict higher levels of burnout and (ii) the impact of perceived COVID-19 stressors on burnout is mediated by resilience. Additionally, in line with the stress-buffering hypothesis and earlier studies showing that social support acts as an effective mechanism to boost resilience (19, 26, 27), we hypothesized that (iii) greater perceived social support predicts lower levels of burnout, and (iv) social support increases individual resilience.

## Methods

### Participants and procedure

The current investigation was part of the larger study examining the impact of COVID-19 on psychological well-being in Singapore. Participants comprised 858 Singapore Residents and Permanent residents aged 21 years and above who had agreed to be recontacted during the first phase of the study (28, 29). Prior to the commencement of the survey, researchers went through an information sheet detailing the study objectives, procedures, potential benefits, risks, confidentiality as well as the participant’s rights to refuse participation. Written consent to participate in the current follow-up study was obtained from each participant. 76% of participants who completed the first study participated in the current study. The current study was approved by the National Healthcare Group Domain Specific Review Board (Ref: 2021/00566).

The study was conducted between October 2021 and September 2022 and coincided with the “Stabilisation” and “Transition” phases wherein the number of community cases stabilised, and safe management measures were relaxed, gradually preparing the nation to transit into endemicity. Even so, social gathering limits, mandatory mask wearing, border control measures and default work from home for many companies were still applicable. Interviews were carried out by trained researchers primarily via the videoconferencing platform “Zoom”. In-person interviews were offered to participants who wished to participate but were not comfortable with the “Zoom” format. Interviews were conducted in English, Malay or Mandarin, based on the language the participant was most comfortable with. The interviews took about 1 h, and participants were compensated SGD40 for their time and effort. A helpline brochure containing a list of organisations providing psychological support was shared with all participants before the survey interview.

### Measures

The measures used in the current study comprised the following:

- Sociodemographic information on age, gender, ethnicity, marital status, employment status, highest educational attainment, parental status, and monthly personal income.
- COVID-19 related stress was assessed using a binary (Yes/No) scale that asked participants about the presence of worry relating to ten items including fear of the self or friends and family contracting the virus, fear of the self or friends and family dying due to the virus, overseas travel restrictions, working from home, restrictions on social gathering, unemployment, having to take unpaid leave and school closure. Total stress was obtained by summing the number of stressors endorsed. The internal consistency of this scale was 0.73.
- The Copenhagen Burnout Inventory (CBI) Personal-related and Work-related subscales (30). The Personal-related burnout scale consists of six items measuring both general physical and psychological exhaustion and was administered to all participants (e.g., How often do you feel worn out?). Items were rated on a 5-point scale where “Always” = 100, “Often” = 75, “Sometimes” = 50, “Seldom” = 25 and “Never/Almost never” = 0. The Work-related burnout scale consists of seven items measuring fatigue derived from work and were administered to those who were currently employed, home-makers and those who had recently been unemployed in the last month. Homemakers and those who were recently unemployed could opt not to answer questions this scale if they felt that work-related burnout was not relevant to them. Three items (e.g., Does your work frustrate you?) used the response scale “To a very high degree” = 100, “To a high degree” = 75, “Somewhat” = 50, “To a low degree” = 25 and “To a very low degree” = 0 while four items used the same response options as the Work-related scale (e.g., Are you exhausted in the morning at the thought of another day at work?’. Total scores on the sub-scales were the average of the scores on the items, with the last item, “Do you have enough energy for family and friends during leisure time?” being reverse coded. A cut-off score of 50 and above on each subscale indicates moderate or higher levels of burnout (31). The CBI has been previously validated and found to have good psychometric properties (32). It has been used widely in Asian settings (33). Confirmatory factor analysis supported the 2-factor structure of the CBI (Personal related and Work-related Burnout; Refer to Supplementary Figure S1). The CBI was also found to discriminate low-resilience and normal-high resilience individuals in this sample using a survey-weighted t-test (*p* < 0.01). In this study, the internal consistency of Personal-related and Work-related subscales as measured using Cronbach’s alpha were 0.87 and 0.89, respectively. The Client-subscale was omitted for this community sample.
- Social support was measured using the 6-item Medical Outcomes Study Social Support Survey (MOS-SSS-6) (34). Respondents were asked a stem question about the level of social support they received from various sources. Each item was answered on a 5-point scale ranging from “None of the time” to “All of the time”, with scores being 1 to 5, respectively. Mean scores across the six items were calculated with higher scores indicating higher levels of social support. The MOS-SSS-6 has been previously validated with satisfactory psychometric properties (34) and had a Cronbach’s alpha of 0.90 in the current study.
- Resilience was measured using the Brief Resilience Scale (BRS), a 6-item instrument that assesses the ability of individuals to bounce back or recover from stress (35). Participants indicated the extent to which they agreed with each statement on a 5-point scale from “Strongly Disagree” to “Strongly Agree”, with scores ranging from 1 to 5, respectively. Negatively worded items were reverse coded, and a score was derived from the mean of the six items. The BRS has been validated with undergraduates in Singapore and shown to have satisfactory psychometric properties (36). The Cronbach’s alpha for the BRS in this study was 0.80.

### Analysis

All analyses performed in our study included post-stratification survey weights to ensure that the results were reflective of the general population. Data were analyzed using STATA S/E version 15 with a two-sided test and a significance level of 5%. Descriptive statistics of the sample were calculated. Categorical variables were represented as weighted percentages and unweighted frequencies while weighted mean and standard deviation were included for continuous variables. Cronbach’s alpha values were calculated for the individual scales to measure the internal consistency. A confirmatory factor analysis was performed on the burnout scale to test its structural validity (Supplementary Figure S1). Multivariable linear regression was conducted on the sociodemographic variables to investigate which factors were significantly associated with burnout. The sociodemographic variables include age, gender, ethnicity, employment status, marital status, highest education attained, monthly personal income and having any children. Path analysis was conducted using Mplus version 8.8 (Muthen & Muthen) to investigate whether resilience mediated the relationship between social support and COVID-19 stressors with burnout. Beta coefficients were standardized using Mplus STDYX output. Age, gender and ethnicity were adjusted for in the mediation model as perceived social support, stress, burnout and resilience have been reported to be significantly associated with these sociodemographic variables in previous studies (37–44). Criteria for the model were selected in accordance with Hu et al. (45); Root mean square error of approximation (RMSEA) <0.05, comparative fit index (CFI) ≥ 0.95, Tucker-Lewis index (TLI) ≥ 0.95, Standardized root mean residual (SRMR) <0.05.

## Results

The sociodemographic profile and classification of the sample by Personal-and Work-related burnout severity are presented in Table 1. Mean scores on the resilience, social support, COVID-19 stress and Personal-and Work-related burnout scales and their respective standard deviation are summarised in Table 2.

**Table 1:** Frequencies and weighted percentages of sociodemographic variables.

	Weighted percentage	Unweighted frequencies
Age groups		
21–34	26.35	312
35–49	29.36	287
50–64	26.60	171
65+	17.69	88
Gender		
Female	48.93	393
Male	51.07	465
Ethnicity		
Chinese	76.48	322
Malay	11.05	190
Indian	7.73	218
Other	4.74	128
Marital status		
Never married	26.82	264
Married/ cohabitation	63.30	535
Divorced/ widowed/ separated	9.88	59
Employment status		
Unemployed	3.55	33
Economically inactive*	21.09	130
Employed/self-employed	75.36	690
Highest education attained		
Below primary	11.87	31
Secondary school	23.56	104
Pre-U/JC/ITE/polytechnic	25.92	293
University and above	38.65	425
Children		
Yes	60.74	487
No	39.26	371
Monthly personal income (SGD)		
Below 2,000	31.99	196
2,000 to 3,999	26.61	242
4,000 to 5,999	18.79	194
6,000 to 9,999	14.97	138
Above 10,000	7.64	78
Burnout-work (*n* = 674)		
None-mild	77.29	506
At least moderate	22.71	168
Burnout-personal (*n* = 858)		
None-mild	81.07	664
At least moderate	18.93	194

^*^Economically inactive group includes retirees, students, and homemakers.

Missing data: employment status *n* = 5, highest education attained *n* = 5 and monthly personal income *n* = 10.

**Table 2:** Weighted mean and SD of variables of interest.

	Cronbach’s alpha	Weighted mean	SD	*n*
Resilience	0.80	3.61	0.60	857
Social support	0.90	69.45	22.00	849
COVID-19 stressors	0.73	3.44	2.61	828
Burnout-personal	0.87	31.36	19.69	857
Burnout-work	0.89	31.71	22.51	673

SD, Standard deviation.

### Sociodemographic factors significantly associated with burnout

The five assumptions of multivariable liner regression (MLR) of (i) linearity, (ii) little/no multicollinearity, (iii) multivariate normality, (iv) no auto correlation and (v) homoscedasticity were met. MLR for sociodemographic variables showed that, age was significantly associated with both Personal-related and Work-related burnout. The coefficients decreased progressively as age increased, indicating that individuals aged 21–35 years were the most burnt-out age group in our study. Those of Malay ethnicity (β = 6.71, 95% CI: −5.58 to 1.78) and those whose highest educational level was secondary school (β = 6.56, 95% CI: 0.62 to 12.51) were significantly associated with higher Personal-related burnout. Those who were unemployed (β = 19.94, 95% CI: 6.41 to 33.74) were significantly associated with higher Work-related burnout. The MLR analyses are summarised in Table 3.

**Table 3:** Multivariable linear regression between sociodemographic factors and burnout.

	Burnout-personal (*n* = 839)				Burnout-work (*n* = 664)			
	β-coefficient	*p*-value	95% Confidence interval		β-Coefficient	*p*-value	95% Confidence interval	
Age groups								
65+ (ref)								
21–34	**17.97**	**<0.001**	**10.27**	**25.67**	**26.68**	**<0.001**	**16.74**	**36.62**
35–49	**15.06**	**<0.001**	**7.7**	**22.42**	**21.58**	**<0.001**	**11.41**	**31.75**
50–64	**7.45**	**0.04**	**0.09**	**14.81**	**9.93**	**0.02**	**1.49**	**18.36**
Gender								
Female (ref)								
Male	−1.9	0.311	−5.58	1.78	1.72	0.44	−2.59	6.02
Ethnicity								
Chinese (ref)								
Malay	**6.71**	**0.01**	**1.84**	**11.58**	3.92	0.17	−1.70	9.55
Indian	2.36	0.23	−1.49	6.19	−0.34	0.88	−4.91	4.23
Others	3.55	0.15	−1.27	8.35	−0.49	0.87	−6.28	5.31
Employment status								
Employed/self-employed (ref)								
Unemployed	7.16	0.08	−0.76	15.06	**19.94**	**0.01**	**6.14**	**33.74**
Economically inactive	6.04	0.11	−1.32	13.38	0.35	0.94	−9.13	9.83
Highest education attained								
Degree and above (ref)								
Below primary	4.85	0.23	−2.99	12.69	2.60	0.54	−5.72	10.92
Secondary school	**6.56**	**0.04**	**0.62**	**12.51**	0.82	0.83	−6.47	8.10
Pre-U/ JC/ ITE/ Poly	1.09	0.59	−2.93	5.11	1.56	0.57	−3.83	6.96
Marital status								
Never married (ref)								
Married/cohab	−2.59	0.42	−8.93	3.75	−4.04	0.21	−10.31	2.23
Divorced/widowed/separated	−3.76	0.40	−12.56	5.04	3.33	0.52	−6.87	13.52
Monthly personal income								
2,000 to 3,999 (ref)								
Below, 2,000	0.03	0.99	−6.57	6.64	2.81	0.52	−5.74	11.36
4,000 to 5,999	2.76	0.25	−1.98	7.49	3.54	0.42	−5.04	12.12
6,000 to 9,999	0.46	0.87	−4.90	5.82	2.38	0.61	−6.86	11.61
10,000 and above	2.81	0.39	−3.53	9.15	8.00	0.16	−3.06	19.06
Have children?								
No (ref)								
Yes	−0.93	0.80	−8.18	6.31	−0.67	0.83	−6.94	5.59

Sample sizes for burnout-personal is different from the burnout-work as the latter was not applicable to some respondents (e.g., students, retirees). Cases with missing data were removed from the analysis. Bold values refer to *p*-value <0.05.

### Mediation analysis

**Figure 1:**
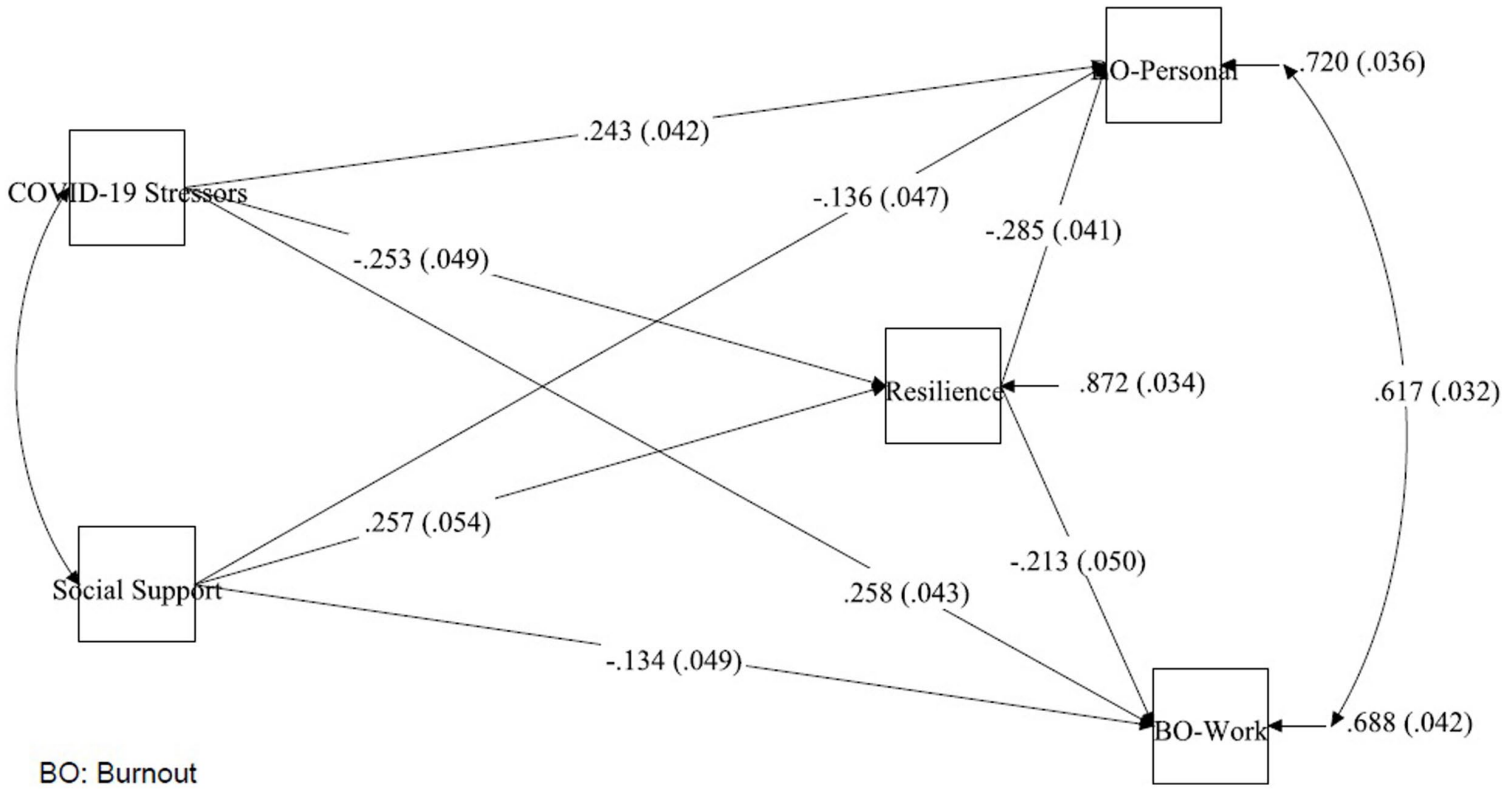
Standardised paths of COVID-19 stressors, social support to burnout, with resilience as a mediator.

Our final path analysis is presented in Figure 1. The model was adjusted for age, gender, and ethnicity. Model fit indices and the criteria of good fit (RMSEA = 0.012, CFI = 0.999, TLI = 0.994, SRMR = 0.018) are presented in Table 4. The direct, indirect, and total effects of the final model are presented in Table 5.

**Table 4:** Model fit indices from the path analysis.

Fit indices	CFI	TLI	RMSEA	SRMR
Results	**0.999**	**0.994**	**0.012**	**0.018**
Criteria	> = 0.95	> = 0.95	<0.05	<0.05

Bold values refer to *p*-value <0.05.

**Table 5:** Standardized effects for the paths between COVID-19 stressors and social support with burnout, mediated by resilience.

	Standardized effects*	SE	*p*-value
Direct effects for burnout work (*n* = 653)			
COVID-19 stressors → BO work	0.258	0.043	<0.001
Social support → BO work	−0.134	0.049	0.007
Direct effects for burnout personal (*n* = 821)			
COVID-19 stressors → BO personal	0.243	0.042	<0.001
Social support → BO personal	−0.136	0.047	0.004
Indirect effects for burnout work (*n* = 653)			
COVID-19 stressors → Resilience → BO work	0.054	0.017	0.001
Social support → Resilience → BO work	−0.055	0.017	0.001
Indirect effects for burnout personal (*n* = 821)			
COVID-19 stressors → Resilience → BO personal	0.072	0.017	<0.001
Social support → Resilience → BO personal	−0.073	0.02	<0.001

*Model was adjusted for age, gender and ethnicity.

### Mediation effects of resilience on COVID-19 stressors

The direct effects of COVID-19 stressors on Personal-related burnout (β = 0.243, *p* < 0.001) and Work-related burnout (β = 0.258, *p* < 0.001) were significant, indicating that greater COVID-19 stressors was associated with a higher burnout. The indirect effects of COVID-19 stressors on personal burnout (β = 0.072, *p* < 0.001) and work burnout (β = 0.054, *p* = 0.001) via resilience were found to be significant as well indicating that resilience partially mediated the relationships between COVID-19 stressors and both Personal-and Work-related burnout.

### Mediation effects of resilience on social support

The direct effects of social support on Personal-related burnout (β = −0.136, *p* = 0.004) and Work-related burnout (β = −0.134, *p* = 0.007) were significant, suggesting that higher social support can lead to lower burnout. The indirect effects of social support on Personal-related burnout (β = −0.073, *p* < 0.001) and Work-related burnout (β = −0.055, *p* = 0.001) via resilience were significant as well, indicating that resilience partially mediated the relationships between social support and both Personal and Work-related burnout.

## Discussion

Twenty-two percent (22%) of the sample reported elevated levels of work-related burnout which was slightly lower than the proportion found among non-clinical staff of a community mental health service assessed in 2019 prior to the outbreak (25%) (46). The percentage of those who reported elevated personal-related burnout in our study was even lower at 19%. While we were not able to identify other studies conducted in the general population for comparison, our mean burnout scores (Personal: 31; Work-related: 31) were lower than those among samples working non-clinical jobs reported in other countries during the pandemic. To illustrate, a study in Thailand among librarians reported Personal and Work-related burnout scores of 44 and 42 (47) while another among teachers in Ireland reported scores of 65 and 61, respectively (48). A possible explanation offered by See et al. who observed lower burnout rates among physicians in Singapore and Hong Kong (31 and 31%) compared to their counterparts in the US (45–55%) pre-pandemic, was that local work culture and values including collectivism, persistence and *guanx*i (respecting social orders and protecting others’ reputation) could have blunted overall self-reports of burnout (31). This explanation however conflicts with the survey findings alluding to the high level of burnout in Singapore compared to other countries (3). Likely, burnout symptoms may have alleviated as our study extended till September 2022 when majority of the safety measures were relaxed.

However, as predicted, higher personal-and work-related burnout was observed among those who experienced greater COVID-19 related stress during the second year of the pandemic. Longitudinal studies have shown that excessive and prolonged stress that is not ameliorated leads to feelings of physical and mental exhaustion, cynicism and depersonalisation, and low personal efficacy, which are the hallmarks of burnout (49). Additionally, the impact of COVID-19 stressors on elevated burnout levels was partially accounted for by reduced resilience, corroborating earlier research (23, 25, 50, 51). Our finding also extends the relevance of this mediational relationship that has largely been documented in workplace settings among frontline staff such as doctors, nurses and police officers to those in the home and community settings.

Resilience has been described as mental fortitude to navigate unpleasant challenges or positive adaptation to adversity (51, 52). The Conservation of Resources (COR) theory asserts that individuals strive to acquire, maintain and protect valuable resources (e.g., finances, health, energy) and experience stress when access to essential resources are threatened. Based on this theory, resilience constitutes a personal resource that supports an individual’s ability to bounce back from negative emotional states, flexibly adapt to the changing demands of stressful experiences, allowing the individual to recoup their resources (53). For instance, in the context of the pandemic, resilient individuals may set boundaries to manage their new work-life arrangements or adjust their financial goals while seeking alternate sources of income. Some attributes of resilient individuals include optimism, hope, enthusiasm, and the ability to develop meaning from hardship (54). The resilience trait thus creates a positive feedback loop that sustains a “gain spiral” preserving an individual’s well-being during challenging times (55).

Our findings further supported the hypothesis that those with higher perceived social support will be less burnt out both on the personal and work fronts and that resilience has a role to play in these relationships. Our findings were similar to that of Shang and Yang (26) who demonstrated that higher social support was associated with lower athlete burnout and this relationship was partially mediated by resilience. Our results espouse assertions that resilience can be augmented by supportive social environments. When dealing with a crisis such as the COVID-19 epidemic, effective social support may reduce respondent’s worries in a similar way that illness support groups do. When respondents had support networks to confide in, their concerns and worries can be better understood and soothed. The individual could also obtain strength, confidence and inspiration from others undergoing similar challenges as them, thereby reducing distress and burnout in the long run (56).

The protective effects of resilience in enhancing well-being and preventing psychological morbidity is well-established and resilience training programmes have gained popularity in the recent years. Presently, resiliency training is a loosely defined set of interventions aimed at enhancing resilience through a range of therapeutic approaches such as mindfulness, stress-management or cognitive behavioral techniques and may feature explicit teaching of emotion-regulation, optimism and self-efficacy (57). Although the lack of standardisation of resilience programmes has been criticised, there is no “one size fits all” formula as different aspects of resilience could be differentially valued based on factors such as nature of the stressor, culture, life-stage etc. Nevertheless, in regard to the pandemic, Kauderer et al. recommended that interventions could include effortfully maintaining positive emotions even in the presence of negative ones (e.g., seeking gratitude for what has not been), reframing negative circumstances (e.g., conceptualising quarantine as a chance to pick up a new skill), maintaining social connectedness (e.g., virtually with restrictive measures) and practicing spirituality (e.g., turning to religion for guidance or engaging in meditation) (57). Moving further upstream, efforts to improve community resilience such as continuous investment in public mental health surveillance and programmes, clear and up-to-date accessible communication between government agencies and the public to reduce anxiety and confusion and cultivating a culture of strong community support and cohesiveness can better prepare societies for future outbreaks (58).

Our study also showed that younger age was associated with both personal and work-related burnout with the 21–35 years age group reporting the highest level of burnout. Various studies have reported comparable age trends (59–61). Huang et al. reasoned that age affects how individuals perceive and cope with stress; as one matures, they acquire traits and psychological capital that improve their resilience towards stressors. They may also become more skilful in rallying support that can buffer the impact of stress (61). Furthermore, the 21–35 years age group are a part of the “sandwich generation” that are caring for both young children and older adult parents, who are more vulnerable to the virus, a point earlier identified by other researchers (59, 62). In addition, those in their 30s are likely to be middle managers who have been reported to be most stressed during the pandemic, as a result of increased complexities in their work that could jeopardise their position in the organisation or career aspirations, in contrast to those 40 years and older who have achieved more stability in their careers (3, 50).

Next, we found that individuals who were recently unemployed demonstrated higher levels of work-related burnout compared to those who were employed. Safety measures that persisted in the second year of the pandemic gave rise to and exacerbated stressful work environments (63). Healthcare sectors continued to have high caseloads whereas industries such as aviation and hospitality were forced to make budget cuts that added to workloads, resulting in reduced job satisfaction (64, 65). Additionally, increased virtual meetings due to remote working and screen time led to “Zoom fatigue” and digital exhaustion (66), while the absence of in-person interaction led to isolation and feeling of lack of support. These factors have been associated with burnout and turnover intentions (64, 65, 67). Thus, it is possible that such factors could have caused individuals in our study to resign, explaining the association between recent unemployment and higher work-related burnout. Indeed, another local survey among 1,002 workers in Singapore aged 16–55 years reported that 46% experienced increased stress, 44% perceived heavier workloads, 33% felt more burnout, 20% felt isolated, 49% realised that they do not like their current job and 24% planned to leave their current employer in the next 6 months (68) mirroring the trend observed in the United States dubbed the Great Resignation where monthly resignations in 2021 were the highest in country’s 20-year history (67). Various studies have emphasized the importance of providing employer, peer and job support to reduce burnout and turnover intentions (69).

Finally, it was observed that those with secondary school education (compared to university) and Malay ethnicity were associated with higher personal burnout. Individuals with lower education levels tend to occupy non-PMET (Professionals, Managers, Executives, Technicians) vocations, that are generally lower paying and face higher job insecurity. Similarly, local reports indicate that those of Malay ethnicity are concentrated in lower rung vocations or in sales and service industries that tend to be most affected by the outbreak (70). These groups may face the impact of the pandemic more severely due to financial instability, higher risk of exposure to the virus, a lack of resources and conducive home environment to manage home-based learning, less help with childcare, and means to afford medical care among other stressors (58), highlighting widening social inequalities during this period.

In Singapore, national reserves and past budgetary savings allowed the government to introduce a series of initiatives swiftly to protect jobs, support households and companies. For instance, the Workfare Special Payment, Self-employed Person Relief Scheme and COVID-19 Support Grant were implemented to provide income support and alleviate financial hardship. Efforts from various government agencies were also aimed at creating jobs and re-skilling workers (58). In contrast, mental health interventions and responses were inadequate and slow, placing a spotlight on investments needed in mental health infrastructure, programmes, and research to enhance and accelerate the public’s psychological preparedness for adversity. Recently, the National Mental Health and Well-being Strategy was launched to tackle mental health issues. The strategy comprises not only an expansion of mental health services but the promotion of well-being through whole-of-society efforts that involve an individual’s microsystem such schools, workplaces, healthcare, and social services. The new strategy also involves identifying varying mental health needs in the population using a tiered care model and emphasizes a preventative approach to improving mental health (71). Continued tracking of the nation’s psychological health is needed to better understand the aftermath of the pandemic and assess the impact of the newly launched strategy.

### Strengths and limitations

The study comprised a representative sample in Singapore and efforts were made to conduct the interviews in local languages (English, Chinese and Malay), using the modality (Zoom or face to face) preferred by respondents. However, as there was no baseline data, the results were analyzed cross-sectionally and causality cannot be inferred. All measures were based on self-report; it is possible that individuals who are burnt out were less likely to take part in survey, thus the data may be an under-representation of burnout levels in the community. Nevertheless, important insights into sections of the population that reported high levels of burnout and the protective effects of social support and resilience on COVID-19 stressors and burnout were identified.

## Data availability statement

The data supporting the conclusions of this article will be made available, upon request to the corresponding author.

## Ethics statement

The studies involving humans were approved by National Healthcare Group, Singapore Domain Specific Review Board (Ref: 2021/00566). The studies were conducted in accordance with the local legislation and institutional requirements. The participants provided their written informed consent to participate in this study.

## Author contributions

ShS: Conceptualization, Investigation, Project administration, Writing – original draft, Writing – review & editing. ET: Formal analysis, Investigation, Writing – review & editing. SaS: Project administration, Supervision, Writing – review & editing, Methodology. YT: Project administration, Writing – review & editing. SG: Project administration, Writing – review & editing. RT: Project administration, Writing – review & editing. PS: Project administration, Writing – review & editing. YZ: Project administration, Writing – review & editing. PW: Project administration, Writing – review & editing. ST: Project administration, Writing – review & editing. MS: Conceptualization, Funding acquisition, Investigation, Methodology, Supervision, Writing – review & editing.
