# Supplementary figures and images for: The protective role of resilience and social support against burnout during the COVID-19 pandemic
Source: Front Public Health. 2024 Apr 30;12:1374484. doi: 10.3389/fpubh.2024.1374484 (PMC11091290; doi:10.3389/fpubh.2024.1374484)

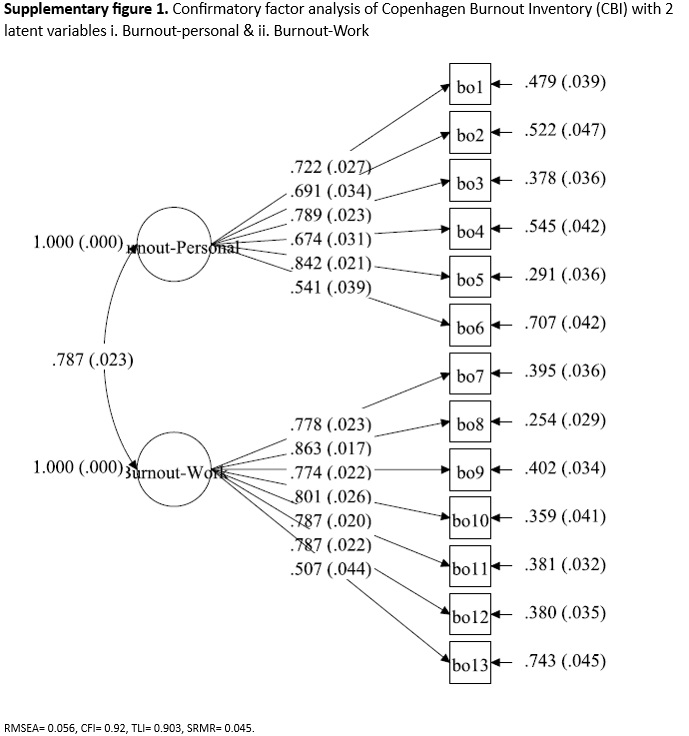

Supplement: Supplementary file 1 [file Image_1.jpg]
